# MedZeroSeg: Zero-shot medical image segmentation via vision foundation models

**DOI:** 10.1371/journal.pone.0344978

**Published:** 2026-03-27

**Authors:** Ronghui Zhang, Min Huang, Rui Li

**Affiliations:** 1 Concord University College Fujian Normal University, Fuzhou, China; 2 College of Electronic Information Science, Fujian Jiangxia University, Fuzhou, China; 3 Smart Home Information Collection and Processing on Internet of Things Laboratory of Digital Fujian, Fuzhou, China; 4 School of Finance and Economics, Jimei University, Xiamen, China; Chengdu University of Traditional Chinese Medicine Wenjiang Campus: Chengdu University of Traditional Chinese Medicine, CHINA

## Abstract

A novel medical image segmentation framework, **MedZeroSeg**, is proposed to address key challenges in the field. Leveraging vision foundation models such as CLIP (Contrastive Language-Image Pre-training) and SAM (Segment Anything Model), it achieves zero-shot segmentation, accurately delineating previously unseen medical images without requiring additional labeled data. This significantly reduces reliance on large-scale annotated datasets. At its core, MedZeroSeg introduces a Dual-Path Feature Extraction Module that captures both fine anatomical details and global contextual information through the integration of local and global perception mechanisms, enhancing robustness against the complexity and variability inherent in medical imaging.Additionally, a Context-Enhanced Hard-Negative Contrast Loss is introduced to enhance contrastive learning by exploiting contextual cues and refining hard-negative sampling, leading to better representations and higher efficiency. The key innovation of MedZeroSeg lies in its ability to leverage generalizable knowledge from CLIP and SAM without any task-specific fine-tuning, making it highly adaptable across different medical imaging modalities. Extensive experiments on three publicly available datasets, including cardiac MRI (ACDC), multi-organ abdominal CT (Synapse), and chest X-ray (COVID-QU-Ex), demonstrate that MedZeroSeg achieves superior results in both zero-shot and weakly supervised segmentation settings, showcasing strong generalization capabilities and minimal data dependency. The framework represents a significant advancement in medical image analysis and opens up promising directions for future research in applying advanced foundation models and innovative learning strategies to healthcare applications.

## Introduction

Medical image segmentation plays a fundamental role in contemporary clinical workflows, facilitating disease diagnosis, treatment planning, and quantitative analysis of pathological structures [1, 2, 3, 4, 5]. Although deep learning-based segmentation models [6–9] have achieved remarkable success in recent years, several longstanding challenges remain unresolved. These include the scarcity of well-annotated datasets, the limited generalization ability of models trained on restricted domains, and insufficient robustness when faced with variations in imaging modalities, acquisition conditions, and patient-specific anatomical differences. As annotation in medical imaging is expensive and time-consuming—often requiring expert radiologists—there is an urgent demand for methods capable of achieving accurate segmentation with minimal or even no task-specific labels while maintaining broad adaptability across diverse clinical environments.

With the emergence of large-scale vision-language models such as CLIP (Contrastive Language-Image Pre-training) [10] and foundation segmentation models like SAM (Segment Anything Model) [11], new opportunities have arisen for zero-shot and cross-domain segmentation [12,13]. These models demonstrate impressive generalization on natural images and have sparked interest in their potential use in medical imaging [14–17,18]. However, transferring such models to medical domains remains nontrivial. The gap between natural and medical images, manifested in modality, specific visual characteristics, subtle pathological cues, and complex anatomical structures—raises ongoing debates regarding how effectively these models can be adapted without large-scale medical data or domain-specific fine-tuning [19,20]. As a result, the feasibility and reliability of zero-shot segmentation in clinical settings remain open research questions.

Another challenge concerns the imbalance between local detail extraction and global contextual understanding. Classical architectures such as U-Net [21], and other CNN-based models [22–24] excel at capturing local features but often fail to encode long-range contextual information, which is critical for distinguishing ambiguous anatomical boundaries or small lesions [25]. Multi-scale feature fusion techniques [26] and attention-based methods [27] partially alleviate this issue, yet they tend to increase computational cost and still rely heavily on annotated data. The tension between preserving fine anatomical detail and capturing global structure remains a central point of discussion in the community.

In parallel, contrastive learning has emerged as a powerful self-supervised paradigm for representation learning [28]. However, traditional contrastive objectives such as InfoNCE [29] often suffer from inefficient or noisy negative sample selection, limiting their discriminative power, especially in the context of subtle medical variations. Recent work on learning from AI-generated annotations [30] has shown promise in reducing annotation burden, yet the integration of such approaches with zero-shot foundation models remains underexplored. The community continues to debate how to incorporate contextual cues, meaningful hard-negative mining, and multi-modal information into contrastive learning to maximize its benefit in clinical applications.

To address these challenges, we propose MedZeroSeg, a novel framework that integrates the strengths of CLIP and SAM to achieve zero-shot medical image segmentation in radiological tasks. By leveraging these models, MedZeroSeg performs accurate segmentation without additional annotations, substantially reducing reliance on large-scale labeled datasets. To enhance the balance between local detail extraction and global context understanding, we propose a Dual-Path Feature Extraction Module (DPFEM). This module combines local and global perception pathways to capture both fine anatomical structures and broader contextual information. The dual-path design enables the model to better comprehend subtle details and overall image layouts, thereby improving segmentation accuracy and robustness. To optimize contrastive learning, we introduce a new loss function named Contextual-Enhanced Hard-Negative Contrastive (CEHNC) Loss. This loss function enhances contrast learning efficiency by utilizing contextual information more effectively and refining the selection of hard-negative samples. Consequently, it improves both the training efficiency and the accuracy of segmentation by enhancing the discrimination between positive and negative samples, allowing the model to learn more discriminative feature representations.

Our approach makes three primary contributions:

1. Zero-shot Medical Segmentation: By integrating the capabilities of CLIP and SAM, our framework achieves zero-shot segmentation of medical images without additional annotations, dramatically reducing dependence on large-scale labeled data.
2. DPFEM: We design a feature extraction module that integrates local and global perception to capture fine anatomical details and rich contextual information, thereby enhancing the model’s capability to process complex medical images.
3. CEHNC Loss Function: We propose a novel CEHNC Loss that enhances contextual information utilization and improves hard-negative sample selection to optimize contrastive learning, thereby boosting training efficiency and segmentation accuracy.

Through comprehensive validation across various medical image segmentation tasks, including cardiac MRI (ACDC) [31], multi-organ abdominal CT (Synapse) [32], and chest X-ray (COVID-QU-Ex) [33], MedZeroSeg demonstrates outstanding performance. It showcases significant potential as an advanced tool for next-generation medical image segmentation, proving its efficacy and versatility in diverse clinical settings.

## Materials and methods

**Fig 1:**
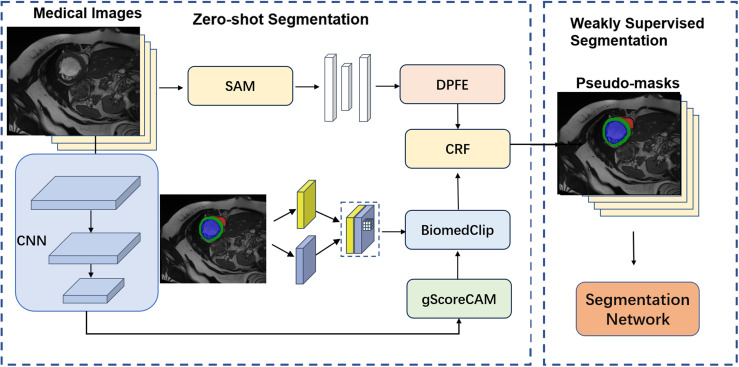
An overview of the proposed MedZeroSeg framework. This figure shows the MedZeroSeg architecture for zero-shot medical image segmentation, highlighting the CEHNC loss for fine-tuning, the DPFEM for feature extraction, and the textual prompt-guided segmentation process.

Fig 1 presents an overview of the Contextual Zero Medical Image Segmentation Network (MedZeroSeg), which consists of three main stages: fine-tuning the BioClip model using the proposed CEHNC loss, performing zero-shot segmentation guided by textual prompts and the DPFEM, and applying weakly supervised segmentation to further refine potential labeling results.

### Context-enhanced hard negative contrastive loss

To address the limitations of current contrastive learning methods in medical image segmentation—particularly their inefficiency in data utilization and inability to distinguish subtle differences—we introduce the CEHNC loss. This newly designed loss function incorporates additional components aimed at improving the model’s discriminative capability and training efficiency.

Unlike traditional contrastive losses such as InfoNCE [29], which typically treat all negative samples equally or use a fixed weighting scheme, CEHNC dynamically adjusts the contribution of each negative sample based on its context and difficulty. Traditional contrastive losses may fail to effectively utilize hard negatives—samples that are visually similar but belong to different categories—which are crucial for enhancing model generalization and learning efficiency.

## Zero-shot and weakly supervised learning

### Adaptive hard negative selection

In contrastive learning, conventional approaches often rely on random or fixed strategies to select negative samples—those semantically different from the anchor (i.e., positive) sample. However, such methods may fail to identify hard negatives, which are visually similar yet belong to different categories and are essential for improving model generalization and learning efficiency. To address this limitation, the CEHNC loss introduces an adaptive mechanism to select hard negatives.

**Fig 2:**
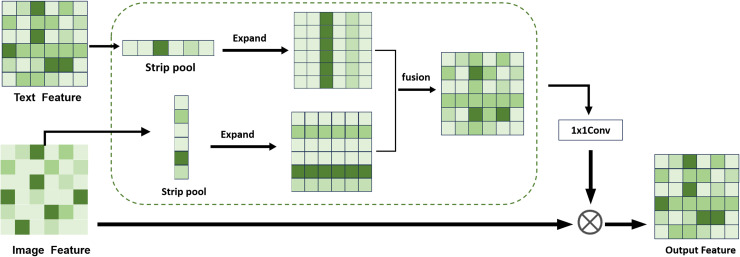
Illustration of the proposed CEHNC loss pipeline. This figure presents the CEHNC loss mechanism, which dynamically selects hard-negative samples and updates difficulty parameters $\beta_{1}$ and $\beta_{2}$ to enhance fine-grained feature discrimination during training.

AS show in Fig 2, the core idea of this mechanism is to dynamically adjust the weight assigned to each candidate negative sample within a training batch. Specifically, at the start of each batch, the similarity between the anchor and all other samples is computed—typically using metrics such as cosine similarity. Based on these similarity scores, the method identifies the most challenging negative samples for the current anchor. Then, considering the overall distribution of the batch, the algorithm adaptively updates two parameters, $\beta_{1}$ and $\beta_{2}$, which control the difficulty curve of the selected negatives. These parameters ensure that sufficient attention is given to hard cases while still allowing for the inclusion of easier samples. As a result, the CEHNC loss enables the model to better focus on distinguishing fine-grained differences, ultimately leading to improved segmentation performance.

### Integration of contextual information

Beyond conventional image-text pairs, the CEHNC loss further incorporates domain-specific contextual information from medical data. This is achieved by integrating relevant clinical metadata, such as diagnostic reports or patient history, into the feature learning process. These additional contextual features are encoded using a dedicated context encoder and subsequently fused with both image and text embeddings before being fed into the contrastive loss function.

To implement this, all auxiliary data sources are first processed through a specialized context encoder, which transforms them into high-dimensional feature representations. These encoded features are then combined with the original visual and textual embeddings to construct a more comprehensive and semantically rich feature space. This integration not only enhances the model’s understanding within each individual modality but also facilitates cross-modal consistency and complementarity.

For example, when a patient’s prior medical records and personal health context are incorporated, the model can better interpret ambiguous findings in a single X-ray image, leading to more accurate identification of pathological conditions. By embedding such rich contextual knowledge, the CEHNC loss significantly improves the system’s ability to recognize complex disease patterns. Moreover, it enhances the robustness and interpretability of the model, enabling predictions that are not only more accurate but also more transparent and clinically meaningful.

### Loss function formulation

The CEHNC loss is designed as a dual-direction contrastive objective that jointly optimizes image-to-text and text-to-image alignment. It is defined as:


$${ℒ}_{\text{CEHNC}}={ℒ}_{\text{img}\to \text{txt}}+{ℒ}_{\text{txt}\to \text{img}},$$ (1)

where each term represents a directional component responsible for pulling positive pairs closer while pushing apart negative ones based on their embedding distances. The revised formulation now includes the positive pair term in the denominator (highlighted in the equations below), following standard contrastive learning conventions. Specifically:


$$\begin{array}{cc} {ℒ}_{\text{img}\to \text{txt}} & =-\frac{1}{M}\sum_{n=1}^{M}\log\left(\frac{e^{\gamma ({𝐳}_{n}^{⊤}{𝐬}_{n})}}{\underset{―}{e^{\gamma ({𝐳}_{n}^{⊤}{𝐬}_{n})}}+\sum_{m\neq n}e^{\gamma ({𝐳}_{n}^{⊤}{𝐬}_{m})}\cdot \omega_{1}({𝐳}_{n},{𝐬}_{m})}\right), \end{array}$$ (2)

$$\begin{array}{cc} {ℒ}_{\text{txt}\to \text{img}} & =-\frac{1}{M}\sum_{n=1}^{M}\log\left(\frac{e^{\gamma ({𝐬}_{n}^{⊤}{𝐳}_{n})}}{\underset{―}{e^{\gamma ({𝐬}_{n}^{⊤}{𝐳}_{n})}}+\sum_{m\neq n}e^{\gamma ({𝐬}_{n}^{⊤}{𝐳}_{m})}\cdot \omega_{2}({𝐬}_{n},{𝐳}_{m})}\right). \end{array}$$ (3)

with **z**_n_ and **s**_n_ denoting the *n*-th normalized embeddings from the vision and language encoders respectively, *M* the batch size, and γ a learnable scaling factor analogous to temperature. To dynamically modulate the influence of negative samples, we introduce two adaptive weighting functions:


$$\omega_{1}(𝐳,𝐬)=\exp\left(-\lambda_{1}\cdot \|𝐳-𝐬{\|}_{2}\right),$$ (4)

$$\omega_{2}(𝐬,𝐳)=\exp\left(-\lambda_{2}\cdot \|𝐬-𝐳{\|}_{2}\right).$$ (5)

where $\lambda_{1}$, $\lambda_{2}$ are hyperparameters that control the sensitivity to hard negatives. These weights suppress the contribution of distant negatives exponentially with respect to their Euclidean distance in the joint embedding space. Unlike temperature scaling (γ), which uniformly scales all similarity scores, the weighting functions $\omega_{1}$ and $\omega_{2}$ apply sample-specific, distance-dependent weights that selectively emphasize hard negatives (samples closer in embedding space). For normalized embeddings, Euclidean distance and cosine similarity are monotonically related ($\|𝐳-𝐬{\|}_{2}^{2}=2(1-{𝐳}^{⊤}𝐬$ for unit vectors), so using Euclidean distance provides a direct hardness signal while maintaining consistency with the cosine similarity used in the contrastive objective. The learnable scaling factor γ is initialized to 1/0.07≈14.3, following the default temperature setting in the original CLIP model, and is optimized jointly with other model parameters during training. In summary, CEHNC loss leverages dynamic weighting and context-aware hard negative mining to enhance the model’s ability to handle subtle differences in medical images, offering significant improvements over traditional contrastive losses.

To fine-tune the BiomedCLIP model [22] using the CEHNC loss function, we utilized the public MedPix dataset containing multiple radiological modalities. In this process, we chose the base version of Vision Transformer as the image encoder, while PubMedBERT [34] was used as the text encoder. To ensure the quality and consistency of the data, we performed a detailed preprocessing work on the MedPix dataset, which included removing all special characters, trimming the front and back margins, and excluding data samples with caption lengths of less than 20 characters. These steps help reduce noise and improve the efficiency of model training.

Next, all images are uniformly resized to a resolution of 224×224 pixels and normalized using the mean and standard deviation values of the RGB channels as defined in the original CLIP model. This normalization strategy ensures better alignment and comparability of visual features across different modalities. Following preprocessing, the dataset is split into training and validation subsets using an 85%–15% ratio, yielding 20,292 images for training and 3,515 for validation. This partitioning strategy guarantees a sufficiently large training set while maintaining a representative validation set for reliable performance evaluation.

During the fine-tuning stage, a relatively low initial learning rate of $1\times 10^{-6}$ is employed, combined with a stepwise learning rate decay of 50%, allowing the model to converge more steadily. Additionally, a batch size of 64 is used throughout training, which provides a balanced trade-off between computational efficiency and accurate gradient estimation. Through these carefully selected hyperparameters and training configurations, we aim to enhance the BiomedCLIP model’s effectiveness in medical image analysis tasks [35].

### Zero-shot segmentation guided by DPFEM-enhanced textual cues

**Fig 3:**
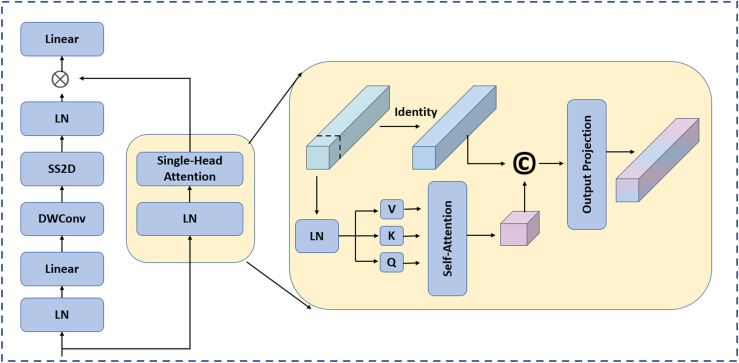
Architecture of the proposed DPFEM. This figure presents the DPFEM architecture, which captures both local details and global contextual information through parallel perception branches and dynamic gated feature fusion to enhance medical image segmentation.

Using the fine-tuned BiomedCLIP model, we propose a zero-shot generalized medical image segmentation strategy. The strategy integrates the XAI technique, gScoreCAM [8], with BiomedCLIP to generate text-related visual saliency maps guided by anatomical or pathological cues. While gScoreCAM has shown superior accuracy and specificity over Grad-CAM [36] for natural images, we extend its use to radiology tasks and further enhance the generated saliency maps through the DPFEM (Fig 3) before post-processing them with Conditional Random Field (CRF) filters.

The gScoreCAM heatmaps are converted to SAM bounding box prompts through the following steps: (1) the heatmap is normalized to [0,1] using min-max normalization; (2) a threshold of 0.5 is applied to obtain a binary mask; (3) morphological closing with a 3×3 kernel is applied to fill small holes and smooth boundaries; (4) the tightest axis-aligned bounding box enclosing all non-zero pixels is computed; (5) this bounding box is provided as the prompt input to SAM for mask generation.

Fig 3 shows an overview of the proposed DPFEM. Our model leverages DPFEM to effectively capture both local details and global contextual information. The operation flow of the framework can be divided into several key steps: first, in the input image preprocessing stage, the original image is processed by an initial convolutional layer, a process that not only initially extracts spatial features, but also appropriately reduces the data dimensionality and lays the foundation for subsequent deeper feature learning. The next step is the local perception branch, where Depth-wise Separable Convolution (DSC) is used, combined with activation functions such as SiLU, which enables the model to retain as much spatial structure information as possible while maintaining high computational efficiency, which is crucial for understanding the subtle but important local features in the image. characteristics of an image. Meanwhile, in the global perception branch running in parallel, the Single-head Self-Attention (SHSA) mechanism is introduced, which allows the model to explore the correlations within the whole image region to better grasp the semantic content of the image as a whole and the interactions between its parts. The last step is dynamic gated attention fusion, where we innovatively combine feature maps from the local perception and global perception branches through a dynamic gating mechanism. This approach dynamically weighs the importance of local and global features based on their relevance and confidence scores, significantly enhancing the model’s ability to handle complex medical images. Unlike traditional fusion methods that simply concatenate or sum features, our dynamic gate adjusts weights in real-time, providing a more nuanced and context-aware feature representation. This dynamic gating mechanism not only improves segmentation accuracy but also boosts the model’s generalization capabilities across diverse imaging modalities.

The comprehensive representation rich in both fine-grained information and macroscopic vision is thus formed, which greatly enhances the model’s comprehension of the complex medical image as well as the expressiveness of the segmentation task.

#### Input image preprocessing.

The input image *I* first undergoes preprocessing through an initial convolutional layer [26]. This step extracts preliminary spatial features and reduces the data dimension for subsequent processing. The output of this step can be represented as:


$$F_{0}=\text{Conv}(I),$$ (6)

where Conv(· denotes the convolution operation.

#### Local perception branch.

This branch utilizes Depth-wise Separable Convolution (DSC) along with an activation function such as SiLU (Sigmoid Linear Unit), to preserve spatial structure information. DSC decomposes a standard convolution into two stages: a depth-wise convolution followed by a point-wise convolution. The process can be formulated as:


$$F_{\text{local}}=\text{SiLU}(\text{Pointwise}(\text{Depthwise}(F_{0})))$$ (7)

where Depthwise(· and Pointwise(· represent the depth-wise and point-wise convolution operations, respectively.

#### Global perception branch.

In parallel with the local perception branch, the global perception branch incorporates a Single-head Self-Attention (SHSA) mechanism, which focuses on learning global dependencies. The SHSA mechanism can be expressed as:


$$F_{\text{global}}=\text{SHSA}(F_{0}),$$ (8)

where SHSA(· represents the single-head self-attention operation. We adopt SHSA rather than multi-head attention for computational efficiency, as our dual-path architecture already provides feature diversity through parallel local and global perception branches. Empirically, SHSA achieves comparable performance to multi-head attention at lower computational cost for our zero-shot segmentation task, while still effectively capturing long-range dependencies between different regions of the image.

#### Feature fusion.

The features from both the local and global perception branches are then fused together. A common approach to fusion is to concatenate the feature maps and pass them through a fully connected layer or another convolutional layer. The fusion process can be described as:


$$F_{\text{fused}}=\text{Conv}([F_{\text{local}},F_{\text{global}}]),$$ (9)

where Conv(· represents the fully connected or convolutional operation, respectively.

We apply a Conditional Random Field (CRF) filter to the fused feature map *F*^fused^ for post-processing, to obtain the initial coarse segmentation result. The CRF filter can smooth the segmentation boundaries and remove noise, thereby improving the segmentation quality. By integrating these two complementary paths, the network is not only able to precisely capture local details but also efficiently learn the global structural information within the images. This overcomes the limitations inherent in traditional CNNs and Vision Transformers (ViTs). Ultimately, the fused features result in a more comprehensive and richer representation, enhancing the model’s performance in tasks such as medical image segmentation. The dual-path design ensures computational efficiency while enhancing the model’s ability to parse complex medical images.

### Weakly-supervised segmentation for potential labeling improvement

On the basis of zero-shot segmentation, we further introduce a weakly supervised learning strategy to improve the accuracy and robustness of the segmentation results. The specific implementation steps are as follows: first, the initial rough segmentation results are obtained by using the fine-tuned BiomedCLIP model and the saliency map generated by gScoreCAM, combined with the DPFEM and the Conditional Random Field (CRF) filter for post-processing. These rough segmentation results are used as pseudo-labels. More accurate pseudo-masks are generated by passing these pseudo-labels to the SAM. These pseudo-masks are used as the results of zero-shot segmentation to provide the basis for subsequent weakly-supervised segmentation training with potential labeling improvements.

In the weakly supervised segmentation training, we use the pseudo-masks generated above as training data. These pseudo-masks are not fully accurate labeling, but they can provide enough information to guide the learning of the model. Meanwhile, we choose residual UNet [23] as the model for weakly supervised training. Residual UNet is an efficient convolutional neural network architecture that can provide high-quality segmentation results while maintaining computational efficiency. During the training process, we use the Cross-Entropy Loss function (CELF) to measure the difference between the model predictions and the pseudo-labeling. Parameter updates are performed using the Adam optimizer with a learning rate of 1E-4 and appropriate decay strategies are set. To increase the generalization ability of the model, we applied various data enhancement techniques such as random rotation, flipping, and scaling during the training process. After weakly supervised training, the model is able to generate finer and more accurate segmentation results. These improved segmentation results not only increase the boundary accuracy, but also reduce noise and mis-segmentation.

## Results

### Experimental setup, datasets, and validation metrics

We evaluate MedZeroSeg on three public medical imaging datasets: ACDC for cardiac MRI, Synapse for multi-organ CT, and COVID-QU-Ex for chest X-ray (pulmonary segmentation). All models are implemented in PyTorch and trained on NVIDIA A100 GPUs with the same hyperparameter settings for fair comparison. All quantitative results are reported as mean ± standard deviation over three independent runs with different random seeds to account for training variability.

Notably, MedZeroSeg performs 2D slice-wise inference for inherently 3D datasets (ACDC and Synapse), due to the 2D-only input requirement of the pre-trained CLIP and SAM models. Consequently, 3D/2.5D inference or volumetric post-processing is not applied. This design choice preserves the strict zero-shot setting without introducing task-specific heuristics.

To evaluate the quality of BiomedCLIP fine-tuning, we performed tests on the ROCO dataset [37], which contains about 7,042 multimodal medical images covering a wide range of clinical cases. We validated the top-1 and top-2 matching retrieval accuracy in both image-to-text and text-to-image directions. For the experiments, we performed five runs, each using a batch size of 50, and ensured diverse combinations of text and images within each batch by randomly shuffling the batch (generating a total of 70,420 rearranged examples). During the fine-tuning process, we compared several different loss functions, including the InfoNCE loss [29], DCL [38], HN-NCE [39], and our proposed CEHNC loss. To ensure fairness, all strategies are trained with the same hyperparameters (temperature τ=0.6, learning rate $=1\times 10^{-6}$). For HN-NCE and CEHNC, we set a uniform difficulty coefficient $\beta_{1}=\beta_{2}=0.15$. In addition, we also consider the pre-trained BiomedCLIP [22], PMC-CLIP [16], and CLIP [10] as the baseline models.

To evaluate the segmentation performance under zero-shot and weakly supervised conditions, as well as the effectiveness of different design components of MedZeroSeg, we selected three public datasets (covering three different imaging modalities) that provide ground truth segmentation labels (cardiac structures, abdominal organs, and lung fields) and are divided into training, validation, and test sets. The details are as follows:

- **Synapse [32]:** Synapse is a public multi-organ segmentation dataset. There are 30 contrast-enhanced abdominal clinical CT cases in this dataset. Following the settings in [40], 18 cases are used for training and 12 for testing. The annotation of each image includes 8 abdominal organs.
- **ACDC [31]:** ACDC is a public cardiac MRI dataset consisting of 100 exams. For each exam, there are two different modalities, and the corresponding label includes left ventricle, right ventricle, and myocardium. The dataset is split into 80 for training and 20 for testing.
- **Chest X-ray (COVID-QU-Ex) [33]:** The COVID-QU-Ex database includes 16,280, 1,372, and 957 chest radiographs (including normal, lung opacities, viral pneumonia, and COVID-19 cases) for training, validation, and testing, respectively.

Based on the above datasets, we conducted a detailed comparative analysis to evaluate the segmentation quality of the initial labels, zero-shot pseudo-masks, and weakly supervised methods generated from the gScoreCAM results after conditional random field processing guided by the DPFEM on each test set. For the ablation study of the zero-shot segmentation task, we paid particular attention to two aspects: first, the effect of BiomedCLIP model fine-tuning on the final segmentation results; and second, comparing the performance differences between two different classes of activation map (CAM) techniques, gScoreCAM and Grad-CAM, in generating segmentation masks. These ablation experiments were all performed on a test subset of each of the three previously mentioned independent datasets.

To ensure the consistency of the experimental conditions, we uniformly used the same SAM architecture, a fixed selection of target layers, a consistent text cueing strategy, and considered only the top 60 most salient channels from each input image for the CAM analysis, regardless of the settings. In addition, several widely recognized quality evaluation metrics were used to measure the performance of the segmentation algorithms throughout the study, including Intersection over Union (IoU),Dice Similarity Coefficient (DSC), and Area Under the Curve of the Receiver Operating Characteristic (AUC). Their formula is as follows.


$$\text{IoU}=\frac{\text{TP}}{\text{TP}+\text{FP}+\text{FN}},$$ (10)

$$\text{DSC}=\frac{2\times \text{TP}}{2\times \text{TP}+\text{FP}+\text{FN}},$$ (11)

$$\text{AUC}=\int_{0}^{1}\text{TPR}(\text{FPR})\;d(\text{FPR}).$$ (12)

By implementing a paired-samples t-test, we were able to scientifically verify whether the observed phenomena and trends were statistically significant. Specifically, when the p-value is less than 0.05, it can be considered that there is a significant difference between the two sets of data, thus supporting our experimental conclusions. Metrics such as HD95 and ASSD were not reported, as they are highly sensitive to small boundary discrepancies and annotation noise, which are pronounced in medical images and exacerbated in zero-shot settings. Therefore, Dice, IoU, and AUC were selected as more reliable and clinically meaningful evaluation metrics.

### Data preprocessing.

Prior to model training and evaluation, comprehensive data preprocessing was applied to ensure data quality and consistency across all datasets. For the **ROCO dataset**, images were resized to 224×224 pixels and normalized using ImageNet statistics. Text captions were lowercased, tokenized using the BiomedBERT tokenizer, and truncated or padded to a maximum sequence length of 77. Duplicate or ambiguous image-text pairs were removed through automated filtering and manual inspection. For the segmentation datasets, we performed modality-specific preprocessing: (1) In **Synapse** and **ACDC**, CT and MRI volumes were resampled to isotropic spacing (1 mm^3^ voxels), intensity-normalized using z-score normalization per volume, and sliced into axial 2D slices for training; (2) For **Lung X-ray**, chest radiographs were resized to 224×224, and pixel intensities were scaled to [0,1]. To reduce bias, all datasets were checked for patient overlap between train/validation/test splits, and data augmentation (random horizontal flipping, rotation $\pm 10^{\circ }$, and brightness jittering) was applied only on the training sets during fine-tuning.

### Quantitative and qualitative results

Table 1 demonstrates the performance of the BiomedCLIP model on the ROCO dataset in cross-modal retrieval tasks including text-to-image and image-to-text after fine-tuning the model using a number of different loss functions. To provide a benchmark comparison, we also list three pre-trained CLIP models as references, namely, the standard CLIP [10], the medical domain-specific PMC-CLIP [16], and the original version of BiomedCLIP [22]. Through a paired McNemar statistical test, we found that the BiomedCLIP model fine-tuned using our CEHNC loss function significantly outperforms the other available loss functions and all pre-trained baseline models. This result demonstrates the effectiveness of the CEHNC loss function in improving cross-modal retrieval accuracy.

**Table 1:** Top-K cross-modal retrieval accuracy for CLIP models on the ROCO dataset.

Model	Version	img → txt (%)		txt → img (%)	
		top-1	top-2	top-1	top-2
BiomedCLIP	Pre-trained	81.37 ± 0.22	92.22 ± 0.29	80.93 ± 0.21	91.74 ± 0.25
	InfoNCE	83.74 ± 0.28	93.90 ± 0.30	85.26 ± 0.23	94.44 ± 0.27
	DCL	84.07 ± 0.20	94.11 ± 0.26	85.42 ± 0.24	94.54 ± 0.22
	HN-NCE	83.96 ± 0.27	94.03 ± 0.21	85.33 ± 0.29	94.55 ± 0.28
	**CEHNC (Ours)**	**84.66 ± 0.25**	**94.93 ± 0.23**	**86.12 ± 0.26**	**95.29 ± 0.31**
CLIP	Pretrained	26.35 ± 0.20	41.37 ± 0.30	25.84 ± 0.15	40.68 ± 0.22
PMC-CLIP	Pretrained	75.19 ± 0.35	86.91 ± 0.18	76.43 ± 0.31	87.80 ± 0.24

Results are reported as mean ± standard deviation over three independent runs. The proposed CEHNC loss achieves the best performance across all metrics. Evaluation is performed on the ROCO dataset with 1,000 image-text pairs in the test set.

Table 2 shows in detail the accuracy assessment of MedZeroSeg under different settings in the zero-shot segmentation task. Specifically, we compare the performance of the model in two cases: first, using pre-trained BiomedCLIP focuses on context-aware hard negative mining; second, the effectiveness of gScoreCAM versus the Grad-CAM method in generating SAM bounding box cues. The experimental results show that the bounding box cues generated using gScoreCAM are significantly better than those of the Grad-CAM method, suggesting that gScoreCAM is able to improve segmentation accuracy more effectively. In addition, by fine-tuning BiomedCLIP using our proposed CEHNC loss function, not only its superior performance across multiple task types and different image modalities was further verified, but also an overall improvement in segmentation quality was observed. This confirms the effectiveness of the CEHNC loss function for enhancing the performance of BiomedCLIP in medical image processing tasks. In Table 2, we also include the MIS-Net method [41], a SAM-based weakly-supervised baseline, for comparison, against which our improvements are clearly demonstrated.

**Table 2:** Comparison of different models and CAM techniques with standard deviation and training time.

Modality	Model	CAM	IoU (%)	DSC (%)	AUC (%)	Time (min)
ACDC	BiomedCLIP	gScoreCAM	55.67 ± 6.23	65.38 ± 5.42	77.86 ± 5.13	18.3
		Grad-CAM	17.42 ± 7.31	23.14 ± 7.65	59.54 ± 7.12	16.8
	MIS-Net	gScoreCAM	56.23 ± 6.01	66.12 ± 7.21	77.98 ± 6.12	19.1
		Grad-CAM	19.01 ± 8.23	23.89 ± 6.78	60.54 ± 5.89	17.6
	Ours	gScoreCAM	**57.31 ± 5.87**	**67.07 ± 7.95**	**78.65 ± 6.01**	18.7
		Grad-CAM	20.07 ± 8.54	24.72 ± 6.38	61.87 ± 5.23	17.1
Synapse	BiomedCLIP	gScoreCAM	48.04 ± 8.14	64.28 ± 5.64	78.64 ± 5.78	21.7
		Grad-CAM	25.44 ± 5.29	32.17 ± 6.41	75.46 ± 8.34	19.5
	MIS-Net	gScoreCAM	49.21 ± 5.56	65.34 ± 6.54	79.87 ± 7.65	22.8
		Grad-CAM	26.14 ± 6.32	32.45 ± 6.34	76.45 ± 5.21	20.1
	Ours	gScoreCAM	**50.30 ± 5.45**	**65.98 ± 6.72**	**80.55 ± 7.92**	22.5
		Grad-CAM	27.05 ± 6.56	32.88 ± 6.17	77.79 ± 5.01	19.9
Chest X-ray	BiomedCLIP	gScoreCAM	46.51 ± 6.78	62.19 ± 7.19	76.95 ± 5.34	15.2
		Grad-CAM	21.39 ± 7.83	35.26 ± 7.49	59.42 ± 6.21	14.0
	MIS-Net	gScoreCAM	48.21 ± 7.45	63.21 ± 5.98	76.98 ± 6.75	15.8
		Grad-CAM	25.14 ± 6.12	37.76 ± 7.21	61.02 ± 5.45	15.3
	Ours	gScoreCAM	**49.14 ± 7.32**	**64.17 ± 5.23**	**77.57 ± 6.89**	15.6
		Grad-CAM	26.02 ± 5.45	38.89 ± 7.62	62.32 ± 5.18	15.1

Results are reported as mean ± standard deviation over three independent runs. Test set sizes: ACDC (*n* = 200), Synapse (*n* = 30), Chest X-ray/COVID-QU-Ex (*n* = 200). Time is reported as minutes per epoch. The substantial performance improvement of gScoreCAM over Grad-CAM can be attributed to their fundamentally different mechanisms: Grad-CAM relies on gradient backpropagation which can produce noisy saliency maps, whereas gScoreCAM directly evaluates channel importance based on activation patterns, providing more focused localization. Our method with gScoreCAM consistently outperforms other approaches across all modalities.

In Table 3, we show in detail the segmentation accuracy of the proposed methods under zero-shot and weakly-supervised settings, with fully-supervised segmentation as a reference benchmark. This comparison helps to comprehensively evaluate the performance of different methods in real applications.

**Table 3:** Performance of various methods compared to fully supervised baseline.

Modality	Model	IoU (%)	DSC (%)	AUC (%)
ACDC	Saliency Maps + CRF	39.78 ± 6.45	51.29 ± 8.13	73.28 ± 5.67
	Saliency Maps + DPFEM + CRF	57.52 ± 7.91^***^	67.49 ± 5.02^*^	78.99 ± 6.15^*^
	Weak supervision-ResUNet	40.99 ± 6.87	58.31 ± 6.71	81.07 ± 6.98
	Full supervision-ResUNet	52.88 ± 7.39	67.06 ± 6.48	**84.38 ± 7.65**
Synapse	Saliency Maps + CRF	38.71 ± 5.37	52.84 ± 5.87	75.52 ± 6.39
	Saliency Maps + DPFEM + CRF	49.91 ± 6.18^*^	66.48 ± 5.54^*^	81.03 ± 7.16^*^
	Weak supervision-ResUNet	41.55 ± 7.56	58.56 ± 6.97	78.10 ± 6.54
	Full supervision-ResUNet	45.62 ± 6.52	62.36 ± 6.81	79.61 ± 5.98
Chest X-ray	Saliency Maps + CRF	34.61 ± 6.98	49.21 ± 7.84	71.66 ± 6.86
	Saliency Maps + DPFEM + CRF	48.85 ± 7.82^***^	64.24 ± 7.87^***^	78.29 ± 5.82^***^
	Weak supervision-ResUNet	75.95 ± 7.43	85.92 ± 7.02	90.59 ± 5.84
	Full supervision-ResUNet	**94.89 ± 5.75**	**97.31 ± 5.53**	**98.22 ± 5.27**

Results are reported as mean ± standard deviation over three independent runs. Statistical significance (two-tailed paired t-test) is assessed against the Full supervision-ResUNet on the same test set. Test set sizes: ACDC (*n* = 200), Synapse (*n* = 30), Chest X-ray/COVID-QU-Ex (*n* = 200). Significance levels: ^***^p < 0.001, ^**^p < 0.01, ^*^p < 0.05. Note: The statistical significance markers indicate comparisons against the fully supervised baseline, not against the weakly supervised method.

For the zero-shot segmentation task, we compare two different ways of generating initial labels: one is based on the initial labels generated by the gScoreCAM saliency map (“saliency map”), and the other is a pseudo-mask generated via SAM (“saliency map + SAM”). The experimental results show that the method combining BiomedCLIP and SAM exhibits significant superiority in all evaluation metrics, significantly improving segmentation quality (p < 0.05). This suggests that by integrating these two techniques, critical regions in the image can be captured more efficiently, thus improving the accuracy and reliability of segmentation.

Under the weakly supervised setting, the performance comparison between zero-shot and weakly supervised methods shows dataset- and metric-dependent trends. For the Chest X-ray dataset, the weakly supervised ResUNet substantially outperforms the zero-shot approach across all metrics (IoU: 75.95 vs. 48.85, DSC: 85.92 vs. 64.24). In contrast, for ACDC and Synapse, the zero-shot method (Saliency Maps + DPFEM + CRF) achieves competitive or superior performance compared to the weakly supervised ResUNet in terms of IoU and DSC. Specifically, on ACDC, the zero-shot method achieves IoU of 57.52 vs. 40.99 for weakly supervised, and on Synapse, IoU of 49.91 vs. 41.55. It should be noted that the statistical significance markers in Table 3 indicate comparisons against the fully supervised ResUNet baseline, not against the weakly supervised method.

Although current fully-supervised deep learning models [23] provide state-of-the-art accuracy in the field of medical image segmentation, our MedZeroSeg zero-shot segmentation method still demonstrates competitiveness on some specific tasks. Specifically, MedZeroSeg outperforms the ResUNet-based fully supervised method in the ACDC and Synapse segmentation tasks. This finding suggests that our method can provide high-quality segmentation results even in the absence of large amounts of labeled data.

However, the fully-supervised method still performs well in the lung radiograph segmentation task, outperforming the zero-shot method both in terms of evaluation metrics such as IoU, DSC, and AUC. This suggests that sufficient labeled data is still a key factor in improving segmentation accuracy in certain application scenarios.

**Fig 4:**
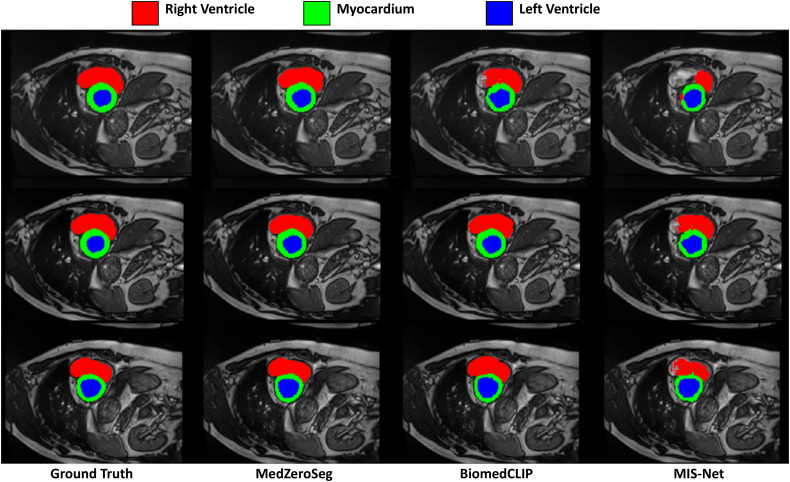
The cardiac MRI segmentation results on the ACDC dataset. Representative qualitative comparison among BiomedCLIP, the SAM-based weakly supervised baseline MIS-Net, and the proposed MedZeroSeg in the zero-shot segmentation setting. For each cardiac MRI slice, the original image, ground-truth label, and predicted masks from the three methods are shown. MedZeroSeg yields more accurate and complete delineations of the left ventricle, right ventricle, and myocardium, with clearer boundaries and fewer spurious predictions than BiomedCLIP and MIS-Net.

Fig 4 presents representative qualitative results for the zero-shot segmentation setting on the ACDC cardiac MRI dataset. Each example includes the original image, the corresponding ground-truth label, and the predictions produced by BiomedCLIP, the SAM-based weakly supervised baseline MIS-Net, and the proposed MedZeroSeg framework. Under the same zero-shot segmentation configuration, MedZeroSeg yields cardiac masks that more faithfully follow the contours of the left ventricle, right ventricle, and myocardium, with more complete coverage of thin myocardial walls and clearer separation between adjacent structures. In contrast, BiomedCLIP often under-segments the ventricular cavities or fails to capture subtle myocardial boundaries, while MIS-Net tends to generate fragmented or overly smooth contours that deviate from the ground truth. Across diverse cardiac views and varying contrast levels, MedZeroSeg produces fewer spurious predictions in the background and better preserves small anatomical details. These qualitative observations are consistent with the quantitative improvements in IoU, DSC, and AUC reported in Table 2, and jointly demonstrate the superiority of MedZeroSeg over BiomedCLIP and MIS-Net for zero-shot cardiac segmentation on ACDC.

**Fig 5:**
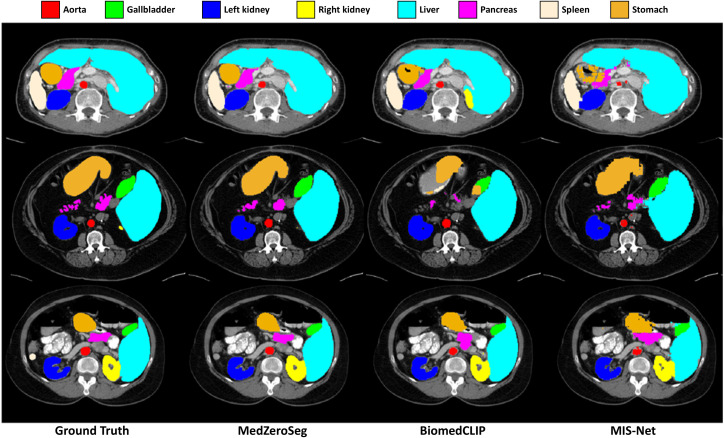
The multi-organ CT segmentation results on the Synapse dataset. Qualitative zero-shot segmentation results comparing BiomedCLIP, MIS-Net, and the proposed MedZeroSeg on representative abdominal CT slices. Each example includes the original image, ground truth annotations, and the corresponding predictions from the three methods. Across multiple abdominal organs, MedZeroSeg produces masks that better align with the ground truth, particularly around challenging boundaries and small structures, while BiomedCLIP and MIS-Net tend to under-segment targets or leak into adjacent tissues.

Fig 5 shows qualitative zero-shot segmentation results on the Synapse multi-organ abdominal CT dataset, again comparing MedZeroSeg with BiomedCLIP and MIS-Net. The examples cover several representative abdominal organs, including both large structures and smaller, shape-irregular targets. Under identical zero-shot segmentation conditions, MedZeroSeg produces organ masks that more closely align with the ground-truth annotations, particularly around complex boundaries and regions with low contrast or ambiguous intensity transitions. BiomedCLIP frequently under-segments target organs or confuses neighboring tissues, while MIS-Net tends to suffer from boundary leakage and missing small structures, leading to incomplete or noisy segmentations. By contrast, MedZeroSeg maintains coherent organ shapes and reduces both false positives and false negatives across different slices and patients. Together with the performance gains summarized in Table 2, the qualitative results in Fig 5 further highlight that MedZeroSeg generalizes more effectively than BiomedCLIP and MIS-Net to challenging multi-organ CT segmentation tasks in the zero-shot setting.

**Fig 6:**
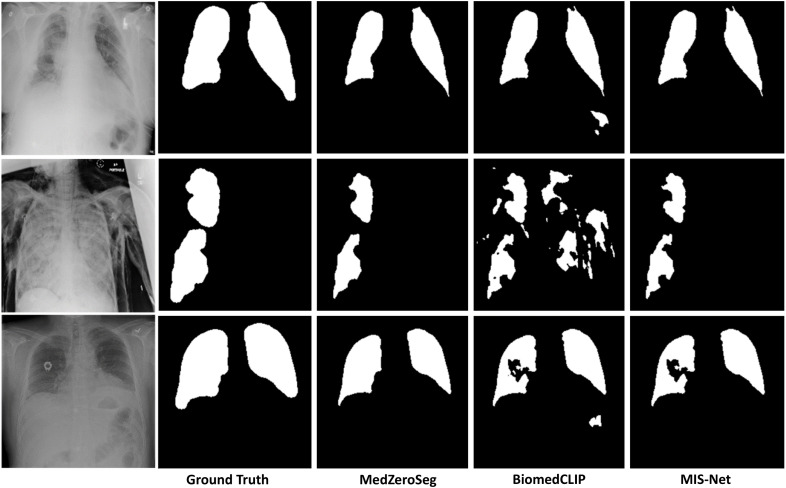
The chest X-ray segmentation results on the COVID-QU-Ex dataset. Representative qualitative comparison of lung or lesion segmentation in the zero-shot segmentation setting using BiomedCLIP, the SAM-based weakly supervised baseline MIS-Net, and the proposed MedZeroSeg. For each chest radiograph, the original image, ground-truth mask, and predicted masks from the three methods are presented. Compared with BiomedCLIP and MIS-Net, MedZeroSeg more accurately captures the extent of pulmonary opacities and preserves anatomically plausible lung boundaries, while reducing false positives in ribs, cardiac regions, and other background structures.

Beyond cardiac MRI and abdominal CT, we further evaluate MedZeroSeg in the zero-shot segmentation setting on the COVID-QU-Ex chest X-ray dataset, which contains chest radiographs from normal, lung opacity, viral pneumonia, and COVID-19 cases. As illustrated in Fig 6, each example shows the original radiograph, the ground-truth lung or lesion mask, and the corresponding predictions from BiomedCLIP, the SAM-based weakly supervised baseline MIS-Net, and the proposed MedZeroSeg. Under identical zero-shot segmentation conditions, BiomedCLIP tends to under-estimate the spatial extent of diffuse pulmonary opacities or miss faint peripheral lesions, while MIS-Net often produces fragmented or overly smooth masks with noticeable leakage into ribs, heart shadows, or mediastinal regions. In contrast, MedZeroSeg generates more contiguous and anatomically plausible segmentations, better delineating the bilateral lung fields and pathological opacities while suppressing background structures. These qualitative findings are consistent with the quantitative results in Table 2, where MedZeroSeg achieves the highest IoU, DSC, and AUC among the three methods on the chest X-ray dataset, further demonstrating its advantage in zero-shot chest X-ray segmentation.

## Discussion

The proposed MedZeroSeg represents an early effort to integrate CLIP and SAM models for generic radiology segmentation tasks. Unlike prior adaptations that combine CLIP or SAM individually for specific medical datasets, our method is specifically designed under a zero-shot setup, where no domain-specific fine-tuning is performed.

By introducing the XAI technique gScoreCAM into the medical imaging domain, our approach establishes a unique saliency-to-prompt conversion mechanism that bridges semantic text embeddings and spatial localization cues. This enables textual cue–based interaction, easy adaptation to unseen data domains or tasks, and efficient utilization of pre-trained models for medical image segmentation. In addition, the proposed dual-path refinement structure and CEHNC loss collaboratively enhance feature alignment between the image and text modalities, achieving both superior segmentation accuracy and robust generalization.

A major contribution of this study is the design of the new CEHNC loss function, which allows more efficient fine-tuning of the BiomedCLIP model than current state-of-the-art loss functions, especially under small batch sizes (see Table 1). Although we have demonstrated the application of the CEHNC loss in unsupervised CLIP model fine-tuning, future work will explore its potential for full model training.

When using BiomedCLIP and gScoreCAM to generate saliency maps, we used simple textual prompts such as “brain tumor” to describe the segmentation task. However, we note that the quality of these saliency maps can be further improved through more sophisticated text prompt engineering, incorporating richer anatomical or pathological descriptors (e.g., shape and location). This opens promising possibilities for interactive radiology education.

As seen in the ablation study, both gScoreCAM and the fine-tuned BiomedCLIP play critical roles in the success of our approach. Weakly supervised segmentation improved accuracy primarily in radiograph-based lung segmentation, while the complex contrast in ultrasound and the inherently 3D nature of the Synapse dataset suggest that volumetric segmentation methods may be more suitable for such data.

**Generalizability limitations.** While MedZeroSeg was evaluated on three publicly available benchmark datasets (ACDC, Synapse, and COVID-QU-Ex), the generalizability of our method to clinical data from different imaging vendors, acquisition protocols, or patient populations remains to be validated. In particular, the COVID-QU-Ex dataset is likely enriched with severe disease patterns such as diffuse bilateral ground-glass opacities commonly observed in severe COVID-19 cases, which may not be fully representative of routine clinical chest radiographs. Additionally, vendor-specific imaging characteristics and center-dependent biases inherent in public datasets may limit the direct transferability of our results to real-world multi-center settings.

**Annotation quality considerations.** The contrasting performance trends observed across datasets may be partially attributable to differences in annotation granularity and precision. The COVID-QU-Ex lung masks are generated using standardized, pixel-level lung field annotations with clear anatomical boundaries, which naturally favor supervised learning approaches. In contrast, ACDC and Synapse involve more complex anatomical structures (cardiac chambers, multiple abdominal organs) where annotations may be coarser or subject to inter-observer variability. Under such conditions, a zero-shot model leveraging pretrained medical priors may appear more competitive relative to supervised models trained to replicate imperfect labels.

Notably, the recent MedSAM [19] has shown excellent performance in medical applications. However, since MedSAM was fine-tuned on a large number of publicly available medical datasets(including our test set), its direct use would violate the zero-shot assumption of our framework. Nevertheless, given the strong baseline performance of SAM in our system, we plan to further explore integrating MedSAM into MedZeroSeg to assess its potential benefits.

Finally, this study was validated across three segmentation tasks and imaging modalities. In future work, we will extend our evaluation to a broader range of medical domains and imaging types to comprehensively assess the generalization ability and practical applicability of MedZeroSeg. External validation on multi-center clinical datasets will be essential to further assess real-world robustness. We hope these efforts will further advance innovation in medical image segmentation and understanding.

## Conclusions

In this study, we introduced MedZeroSeg, a zero-shot medical image segmentation framework that leverages the complementary strengths of CLIP and SAM. Without any domain-specific fine-tuning, MedZeroSeg achieves accurate segmentation across multiple modalities, effectively reducing reliance on large-scale annotated datasets. Our design integrates a DPFEM to capture both local anatomical details and global contextual information, and employs a novel CEHNC Loss to enhance contrastive learning through context-aware hard negative selection. Together, these components enable more robust and efficient feature alignment between image and text modalities. Experimental results on diverse public datasets, including ultrasound, MRI, and X-ray, demonstrate that MedZeroSeg delivers competitive segmentation accuracy and strong cross-domain generalization. While promising, our current framework remains limited by its 2D design, prompt dependency, and potential bias from SAM-based mask proposals. Future work will focus on extending MedZeroSeg to 3D volumetric reasoning, adaptive prompt learning, and uncertainty estimation, as well as validating it on multi-center clinical datasets to further assess real-world robustness and generalizability. In summary, MedZeroSeg provides an efficient, extensible, and annotation-free solution for medical image segmentation, contributing to the broader adoption of foundation models in clinical imaging analysis [20, 25, 18].
